# Supplementary material for: A novel parameterized neutrosophic score function and its application in genetic algorithm
Source: PeerJ Comput Sci. 2024 Jul 11;10:e2117. doi: 10.7717/peerj-cs.2117 (PMC11637011; doi:10.7717/peerj-cs.2117)
Supplement: Supplemental Information 2 [file peerj-cs-10-2117-s002.docx]

*We have provided the raw data summarized in Table 1. After the original data is calculated based on statistics, the table of comparison results in the paper can be obtained. The table of the raw data is as follows:*

|  | Iterations of PNO–PGA | CPU time of PNO–PGA | Iterations of GA | CPU time of GA |
| --- | --- | --- | --- | --- |
| 1 | 398.2632859 | 456 | 389.6123304 | 157 |
| 2 | 404.8126836 | 340 | 437.7019768 | 200 |
| 3 | 412.4543903 | 412 | 469.4698617 | 268 |
| 4 | 410.0422761 | 394 | 389.0721757 | 265 |
| 5 | 415.1748598 | 461 | 436.2190692 | 250 |
| 6 | 412.3403645 | 431 | 434.9040275 | 218 |
| 7 | 401.0981188 | 393 | 409.9557796 | 280 |
| 8 | 400.4870722 | 354 | 400.9136899 | 198 |
| 9 | 408.9079559 | 437 | 399.8252821 | 280 |
| 10 | 397.357105 | 424 | 403.5300288 | 256 |
